# Commentary: Cardiac care during the coronavirus disease 2019 pandemic: Competing constraints

**DOI:** 10.1016/j.xjon.2021.06.009

**Published:** 2021-06-10

**Authors:** Michael J. Reardon

**Affiliations:** Department of Cardiovascular Surgery, Houston Methodist Hospital, Houston, Tex

**Figure:**
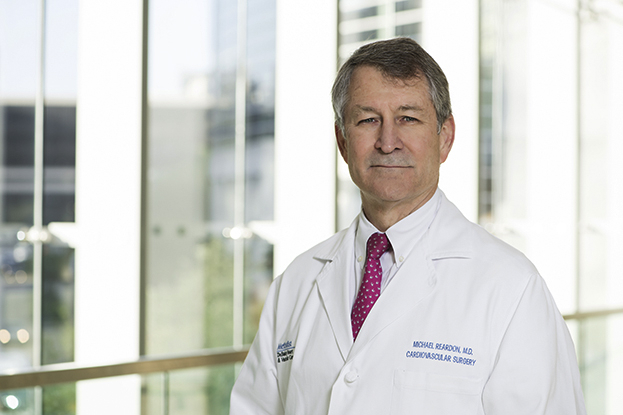
Michael J. Reardon, MD

**Central Message**

Cardiac surgery was limited during the COVID-19 pandemic. TAVR had better 2-year survival unless a patient's risk of contracting COVID-19 during the hospitalization exceeded about 50%.

See Article page 63.

The coronavirus disease 2019 (COVID-19) pandemic caused by the severe acute respiratory syndrome coronavirus 2 has had a devastating effect on the world. As I write this, the COVID-19 statistics for today (coronavirus.jhu.edu) show worldwide cases stand at 172,629,689 and deaths at 3,714,014. In the United States alone, the case count is 33,346,842 and deaths are 597,003. This has affected all of us in both personal and professional ways. We have watched friends, neighbors, and colleagues contract the virus and sometimes die. Sadly, we will never regain those we have lost. In a less drastic, but still difficult way, we have watched as local restaurants closed to in-person dining, schools closed, and in-person medical meetings stopped—only a few of the many changes we all underwent in our lives. As difficult as these were, I watched our communities take on these challenges with expanded takeout service in restaurants, virtual school classrooms, and virtual medical meetings. As we hope for the end of this pandemic, which seems in sight, it is reasonable to ask how this has influenced our ability to deliver care as cardiothoracic surgeons and what this has meant to our patients. During the initial surge, my state, Texas, banned elective cases for a period. Even before this mandate, my institution, in an attempt to conserve personnel, places to operate, and care for patients and protective gear, had already severely limited cardiothoracic surgery procedures. All of our medical intensive care units (ICUs) became COVID ICUs. All intensivists who normally worked in cardiovascular surgery ICUs became full-time COVID-19 intensivists and were moved out of our cardiovascular surgery ICU. The cardiothoracic surgery faculty took over the running of the cardiovascular surgery ICU as in-house ICU physicians in 12-hour shifts for 2 months. This was a drastic change for our department, and as the surgical director of structural heart, I saw both our open and transcatheter valve case numbers plummet. Were the decisions we made correct? Did they lead to the optimal outcomes possible during a pandemic with the competing risk of death from cardiothoracic disease versus death from COVID-19? Freno and colleagues^1^ attempt to answer this challenging question for patients with symptomatic severe aortic stenosis.

Freno and colleagues^1^ look at patients with symptomatic severe aortic stenosis at low or intermediate risk planned for transcatheter aortic valve replacement (TAVR) but delayed by the pandemic. They created a decision tree for the competing risks of aortic stenosis versus COVID-19 and looked at short-term and 2-year survival based on prompt versus delayed TAVR. The discussion of the assumptions that go into this risk model were robust, too lengthy to cover here and largely out of my wheelhouse. Despite any questions, I believe the authors did a good job of modeling this with the limited knowledge available for COVID-19. They defined delay as a 6-month delay, and I find that longer than I would expect, even during the pandemic, for most institutions and 3 months may well have been a more reasonable number. The main finding is not overly surprising in general scope, but does add some knowledge regarding what your risk of getting COVID-19 during hospitalization would need to be to justify waiting 6 months for a TAVR procedure. Immediate TAVR had improved 2-year survival unless the patient's risk of contracting COVID-19 during a hospital stay exceed about 50% (55% for intermediate-risk patients and 47% for low-risk patients). Based on the number of COVID-19 cases I saw occurring new during a hospitalization in my institution, a 50% rate seems exceedingly unlikely. The authors conclude that prompt TAVR was appropriate as long as local health care resources were not overly constrained.

**Figure 1:**
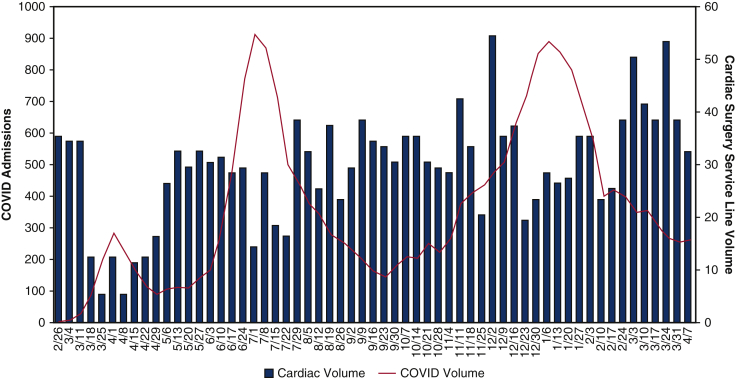
Coronavirus disease 2019 (*COVID-19*) cases and cardiac surgery cases in the Houston Methodist Hospital system. The *redline* is the total number of COVID-19 cases using the *y*-axis. The *blue bars* are the total cardiac surgery cases by week using the *y*-axis. The *x*-axis is by week.

TAVR at our institution was defined as urgent-elective and not elective surgery. The use of an ICU bed occurs < 5% of the time and mean length of stay is 1 day. It would seem that prompt TAVR would make sense because these patients have minimal contact with other patients during this short stay and our TAVR volumes should have held steady. Unfortunately, there are other factors that are difficult to model. During the height of the pandemic, family members were not allowed to stay with patients during their hospitalization and even as restrictions eased, the number of family members who could be with a patient was severely limited. Many of our patients choose to delay their TAVR procedure out of the fear of not having family at their bedside. Additionally, both patients and physicians were learning about a new disease that was initially unpredictable due to lack of knowledge. In our system, we saw 3 distinct surges of COVID-19, and cardiac surgery was affected differently with each surge (Figure 1). The initial surge was the smallest in patient numbers but the most restrictive of cardiac surgery. This occurred because we were underprepared with both resources and knowledge. The additional surges were both larger but had successively less influence because physicians were better prepared with resources and knowledge and patients became less afraid to enter a hospital for treatment. I fully agree with the conclusions drawn by the authors and salute their taking on this difficult challenge. The difficulty still occurs in the difficult-to-measure confounding factors such as patient fear of hospitals during the pandemic or distress at not having family present. Finally, the constraint on resources includes both physical things such as people, places and, protective gear but also intangible issues like knowledge. We as a field have done an outstanding job of reducing these constraints and are now ready to offer TAVR based on medical need and patient desire as the only important factors.
